# Acceptability of point-of-care viral load testing to facilitate differentiated care: a qualitative assessment of people living with HIV and nurses in South Africa

**DOI:** 10.1186/s12913-020-05940-w

**Published:** 2020-11-25

**Authors:** Lindani Msimango, Andrew Gibbs, Hlengiwe Shozi, Hope Ngobese, Hilton Humphries, Paul K. Drain, Nigel Garrett, Jienchi Dorward

**Affiliations:** 1 Centre for the AIDS Programme of Research in South Africa (CAPRISA), University of KwaZulu–Natal, Durban, South Africa; 2 Gender and Health Research Unit, South African Medical Research Council, Pretoria, South Africa; 3 Centre for Rural Health, School of Nursing and Public Health, University of KwaZulu-Natal, Durban, South Africa; 4 Ethekwini Municipal Health Department, Durban, South Africa; 5 Department of Global Health, Schools of Medicine and Public Health, University of Washington, Seattle, USA; 6 Department of Medicine, School of Medicine, University of Washington, Seattle, USA; 7 Department of Epidemiology, School of Public Health, University of Washington, Seattle, USA; 8 Discipline of Public Health Medicine, School of Nursing and Public Health, University of KwaZulu-Natal, Durban, South Africa; 9 Nuffield Department of Primary Care Health Sciences, University of Oxford, Oxford, UK

**Keywords:** Point-of-care, Viral load testing, HIV, Differentiated care, Qualitative, Acceptability

## Abstract

**Background:**

Providing viral load (VL) results to people living with HIV (PLHIV) on antiretroviral therapy (ART) remains a challenge in low and middle-income countries. Point-of-care (POC) VL testing could improve ART monitoring and the quality and efficiency of differentiated models of HIV care. We assessed the acceptability of POC VL testing within a differentiated care model that involved task-shifting from professional nurses to less highly-trained enrolled nurses, and an option of collecting treatment from a community-based ART delivery programme.

**Methods:**

We undertook a qualitative sub-study amongst clients on ART and nurses within the STREAM study, a randomized controlled trial of POC VL testing and task-shifting in Durban, South Africa. Between March and August 2018, we conducted 33 semi-structured interviews with clients, professional and enrolled nurses and 4 focus group discussions with clients. Interviews and focus groups were audio recorded, transcribed, translated and thematically analysed.

**Results:**

Amongst 55 clients on ART (median age 31, 56% women) and 8 nurses (median age 39, 75% women), POC VL testing and task-shifting to enrolled nurses was acceptable. Both clients and providers reported that POC VL testing yielded practical benefits for PLHIV by reducing the number of clinic visits, saving time, travel costs and days off work. Receiving same-day POC VL results encouraged adherence amongst clients, by enabling them to see immediately if they were ‘good’ or ‘bad’ adherers and enabled quick referrals to a community-based ART delivery programme for those with viral suppression. However, there was some concern regarding the impact of POC VL testing on clinic flows when implemented in busy public-sector clinics. Regarding task-shifting, nurses felt that, with extra training, enrolled nurses could help decongest healthcare facilities by quickly issuing ART to stable clients. Clients could not easily distinguish enrolled nurses from professional nurses, instead they highlighted the importance of friendliness, respect and good communication between clients and nurses.

**Conclusions:**

POC VL testing combined with task-shifting was acceptable to clients and healthcare providers. Implementation of POC VL testing and task shifting within differentiated care models may help achieve international treatment targets.

**Trial registration:**

NCT03066128, registered 22/02/2017.

**Supplementary Information:**

The online version contains supplementary material available at 10.1186/s12913-020-05940-w.

## Background

The World Health Organization (WHO) recommends viral load (VL) testing to monitor antiretroviral therapy (ART) effectiveness [1]. However, in many low and middle-income countries (LMICs), VL testing remains challenging due to insufficient laboratory infrastructure, long turnaround times and poor management of results [2, 3]. De-centralised point-of-care (POC) VL testing may help increase coverage and overcome these operational challenges [4]. Additionally, POC testing may improve treatment outcomes and increase efficiency by allowing rapid adherence counselling for clients with high VLs, and referral into streamlined, differentiated care services for those virally suppressed [5]. These differentiated care services aim to cater for the specific needs of PLHIV and promote health system efficiency [6]. Differentiated ART delivery models for well clients include task-shifting (provision of ART by less highly trained healthcare workers) [7], less frequent appointments [8], community adherence clubs [9], and decentralising ART delivery to local pharmacies and collection points [4]. POC VL testing could accelerate triage into differentiated ART delivery services [10].

We performed the Simplified TREAtment and Monitoring study (STREAM), the first randomized trial of POC VL testing and task shifting to nurses. In STREAM, POC testing increased the availability and speed of VL results, leading to improved VL suppression and retention in care [11]. However, we are not aware of any published work assessing the acceptability of POC VL within differentiated care models. Existing literature assessing other POC assays, such as CD4 count testing and POC VL for early infant diagnosis, has found these approaches to be acceptable to patients [12, 13] and to improve clinical outcomes [14, 15]. Qualitative research highlights benefits such as providing results quickly, improving understanding of results, and reduced costs and referrals [16]. However, barriers to implementation have also been described including human resource shortages, high patient volumes and the need to adapt clinical work flows and maintain quality assurance [17, 18]. Further, POC testing may not be acceptable because it increases the burden of work in clinics where busy members of the clinical team have to do the testing [19]. Regarding differentiated care, qualitative assessments of task shifting from doctors to professional nurses have found this to be acceptable [20], but there is little data regarding task shifting to enrolled nurses. Task shifting may not be acceptable if it is considered unsafe for less trained healthcare workers to look after PLHIV, and the additional workload could be seen to be burdensome [21].

In this study, we aim to use client and nurses’ perspectives in the STREAM study to assess the acceptability of POC VL testing and task-shifting from a professional nurse to an enrolled nurse as part of a model of differentiated HIV care.

## Methods

### Study setting

We undertook a qualitative study within the STREAM trial [6], in which 390 adults living with HIV and on ART were randomized to receive either POC VL and CD4 count testing versus standard-of-care laboratory-based testing. POC VL testing and associated clinical care was performed by research staff, using the Xpert HIV-1 VL assay on the GeneXpert platform (Cepheid, Sunnyvale, CA, US), which was placed in an on-site POC laboratory. In the POC arm, clients with a suppressed VL could receive care from an enrolled nurse (2 years training, Registered Nurse equivalent in the US), rather than a more highly qualified professional nurse (the standard care provider in the South African HIV programme with 4 years training, Nurse Practitioner equivalent in the US). In both arms, clients with viral suppression after 6 months in the study could be referred to the Centralised Chronic Medication Delivery and Distribution system (CCMDD), a national differentiated care program in which stable clients collect pre-packed ART from pharmacies and community pick-up points [22]. The study was undertaken at the Centre for the AIDS Programme of Research in South Africa (CAPRISA) eThekwini Clinical Research Site and the Prince Cyril Zulu Clinic, which provides HIV, tuberculosis, sexual health and primary care services for people in Durban, KwaZulu-Natal, South Africa [4, 23].

### Study design

We conducted semi-structured interviews and focus group discussions (FGDs) between March and August 2018 to establish in-depth individual and collective perspectives from clients regarding POC VL testing and task-shifting. We used a purposive sampling frame to recruit participants from the POC testing and standard-of-care arms, with suppressed and unsuppressed VL results, and who were seen by professional or enrolled nurses (Table 1). We recruited participants from the standard-of-care arm in order to assess their experiences and compare the existing model of care and the study intervention. Eligible participants had exited the STREAM trial and were willing to participate in a qualitative study. Nurses who were involved in the STREAM trial and provided consent were also recruited for interviews. Clients and nurses were recruited face-to-face or telephonically.

**Table 1:** Client in-depth interview and focus group discussion sampling frame

		POC clients	SOC clients	Total
		Target	Target	
**Viral load result**	**Care provider**			
**< 1000 copies/ml**	Enrolled nurse and/or CCMDD	5	5^a^	10
	Professional Nurse	5	5	10
**> 1000 copies/ml**	Professional Nurse	5	5	10
Total		15	15	30

^a^Clients in the standard of care arm with suppressed viral loads were eligible for referral into CCMDD but were not seen by an enrolled nurse

*CCMDD* Centralised Chronic Medication Dispensing and Distribution programme; *POC* point-of-care; *SOC* standard-of-care

### Data collection

Interviews and FGDs were conducted at the research clinic using open-ended topic guides encompassing questions about POC VL testing, differentiated care and task-shifting (Additional file 1). These topic guides were developed through collaboration between the research team and the study nurses. FGDs involved 5–10 participants. Interviews and FGDs were conducted in English or IsiZulu by a trained research assistant. LM (male) was not known to participants. HS (female) had recruited participants into the STREAM trial but was not involved in their clinical care. During the FGDs one research assistant facilitated while the other took notes. Audio-recordings were subsequently transcribed and translated into English. We conducted interviews and FGDs until theoretical saturation was reached. Interviews lasted 30–60 min and FGDs 60–80 min. Due to practical considerations in the trial, we first completed interviews, and then conducted FGDs.

### Data analysis

After data collection had finished, we (LM, JD, AG) performed data coding and analysis using NVivo 12 software (QSR International, Melbourne, Australia), employing a thematic content analysis [23] To do this, LM and JD developed a codebook using predefined themes based on the broad topics we asked during the interviews and FGDs (i.e. task shifting, point-of-care testing and differentiated models of care). As coding under these broad topics occurred, we discussed emergent sub-themes and integrated them into the existing themes or, if necessary, created additional themes to reflect the emergent findings (Additional file 2). Coding and analysis was predominantly conducted by LM, supported by JD and a senior qualitative researcher (AG) and there was continual discussion among the team about the themes and sub-themes as coding occurred. Participants did not review the analysis.

### Ethics

The study received ethical approval from the University of KwaZulu-Natal Biomedical Research Ethics Committee (BE648/17) and the University of Washington Institutional Review Board (STUDY00001466). Written informed consent was obtained from all participants. To ensure anonymity and confidentiality, participant’s identifying information was not transcribed and any names and identifying descriptions mentioned in the audio recordings were given fictitious names and descriptions.

## Results

Fifty-five clients on ART participated in the study (25 interviews and 4 FGDs, only one participant was both interviewed and in an FGD). Median age was 31 years, 56% were women and overall demographics (Table 2) were similar to the STREAM study population [11]. We also interviewed 8 nurses (median age 39, 75% women), of whom 3 cared for intervention clients and 5 for standard-of-care clients (Table 2).

**Table 2:** Participants demographic and clinical characteristics

Clients			
		Frequency (*N* = 55)	%
Age	Median (IQR)	31 (27–37)	
Sex	Male	24	43.6
	Female	31	56.4
Arm	Standard of care	18	32.8
	Intervention	37	67.3
Have you disclosed your HIV status to anyone?	Yes	52	94.6
	No	3	5.5
Is your travel time to clinic > 1 h?	Yes	7	12.7
	No	48	87.2
Educational level	None/primary/did not pass secondary school	16	29.0
	Passed secondary school	39	70.9
Income (South African Rands)	< 1000 (70 USD)	23	41.8
	1000–4000 (70–280 USD)	20	36.4
	> 4000 (280 USD)	12	21.8
Number of children	None	10	18.1
	1 or more	45	81.8
VL > 1000 copies/ml	Yes	6	10.9
	No	49	89.1
Seen by an enrolled nurse?	Yes	22	40.0
	No	33	60.0
Collected ART in CCMDD	Yes	32	58.2
	No	23	41.8
**Healthcare Providers**			
		Frequency (*N* = 8)	
Age (years)	Median (IQR)	39 (36–42)	
Sex	Male	2	25.0
	Female	6	75.0
Profession	Professional nurse	4	50.0
	Enrolled nurse	4	50.0
Study arm	Intervention	3	37.5
	Standard of care	5	62.5

*IQR* interquartile range

### Practical benefits

Participants expressed several practical benefits of POC VL testing (Table S1). A single visit encompassing blood testing and review of results meant clients didn’t have to attend clinic multiple times, saving time, days off work and money. As one client said:

*“I waited for 2 hours … and indeed I received all my results. I didn’t leave and come back another day. I got everything the same day.”* (POC female client, 20–30).

In the context of long waiting times at clinics, clients felt waiting 2 h for POC results was acceptable, because it expedited clinical management decisions and enabled them to assess their health progress immediately:

*“It helped me a lot, because everything was done on the day. I didn’t wait for the next month to see my results and start the new [regimen]. Even now, I am waiting for my results; I want to see how I have been doing for the past month in terms of taking treatment, [I want to see] whether I am improving or not … it’s better if everything is done today.”* (POC male client, 20–30).

Several clients and nurses highlighted difficulties employed people had balancing work commitments with regular clinic attendance. POC VL testing reduced the number of days patients needed to take off work (Table S1), which may improve adherence, as one nurse outlined:

*“The patient comes into the clinic, it’s a one clinic visit. They can have their viral load done and if they are virally suppressed, they can have their two months’ supply, or they can have their 6 months’ supply... [at CCMDD]. Everything done in one day. They only have one day off work, because work … sometimes a lot of patients’ default because of work. Work demands them to be there and the clinic demands them to come to clinic.”* (Healthcare provider).

However, there was one client who described being unable to wait for the POC results because of work commitments:

*“I took them [point-of-care bloods], but then I didn’t have time to wait. I said: ‘I am going to work, I won’t be able to wait.”* (POC male client, 30–40).

Most clients and nurses mentioned that POC VL testing also saved clients’ money as they did not have to travel so often to the clinic:

*“I even save money that I use for transport. If I take bloods today and you tell me that I should come back after two weeks, I will need to come back again and pay another transport fare to come here [to the clinic]. Whereas I can wait two hours and get my results and leave afterwards.”* (POC female client, 40–50).

Some clients highlighted that with laboratory testing, their results got lost or they did not get their results at their next visit (Table S1). They felt POC testing could help prevent this:

*“I think it would be better taking the viral load and getting the results on the same day … rather than coming back [for results] next time … because when I come next time, I have some more issues, so I don’t have time to ask for my bloods … “How is my viral load?” you know? But if you wait, wait for the [POC] results... you get your results.”* (Standard-of-care male client, 30–40).

*“The nice thing … is that your results come back same day. [With standard laboratory testing] sometimes when you come, they will tell you that the results are lost and now you must repeat the bloods. But [with POC testing] you sit and wait for them and everything is finished” (POC client, focus group).*

### Meaning of results

Clients described how the immediacy of POC VL results enabled them to demonstrate to themselves, and nurses, they were taking the treatment, motivating ongoing good adherence:

*“We knew that when we go for bloods I would come back [home] knowing what is happening with my life and … that would encourage us to take medication on time”* (POC female client, 20–30).

Clients also highlighted that POC results reduced the anxiety of waiting for results and helped them to monitor the treatment’s effect on their health. One client explained:

*“They take bloods from you and then you wait for your viral load and CD4 count results and know how it is going, and how the treatment is treating you … [With standard laboratory testing] you wait three months before knowing whether you CD4 count is dropping or increasing … You worry a lot not knowing how your viral load is and how your CD4 count is. It’s better to know that today they took the bloods and my CD4 count has dropped and I need to fix this and that”* (POC male client, 20–30).

The contemporaneous monitoring provided by POC testing was also perceived to encourage modification of behavior for those who engaged in activities that might affect their health:

*“It’s good to leave knowing your results because we smoke, we drink and sometimes we engage in unprotected sex … you see all those things you can track that this weekend you were out drinking and the last month you came your CD4 count was high and now it is low, which means the things you do are affecting your health.”* (Client, male, focus group).

Similar to the quote above, several clients focused more on their CD4 count (Table S1), but they learned about the importance of VL testing through receiving POC results:

*“I only knew that my CD4 count is fine and the only important thing to know is a CD4 count until the nurse told me that my viral load is suppressed, and I asked her what a viral load is, and she explained to me. Then I had an understanding that it should be low and my CD4 count must be high.”* (Client, female, focus group).

### Differentiated care

Nurses felt POC VL testing was beneficial as it improved the availability of results and enabled them to provide differentiated care to clients. Clients with high VL could be offered adherence counselling and switch ART regimens earlier if indicated:

*“POC VL testing is good because you give the results at the same time, and you explain them to the patient at the same time. We are also able to counsel them at the same time if the viral load is abnormal and if they need to be switched, they are switched earlier before it’s too late.”* (Healthcare provider).

For virally suppressed and stable clients POC VL testing could assist them to be quickly referred to community-based ART pick-up points to collect their treatment there.

*“Referring them to CCMDD earlier... it helps, because when the [POC] results come, and they are suppressed, we are able to refer them at the same time to the pick-up point that is nearer to them”* (Healthcare provider).

Clients understood referral to the differentiated model of care as a reward for good adherence, while those with unsuppressed VL would continue routine clinic visits.

“*… so it [CCMDD] was more like an incentive that I did well and as a reward you can collect [ART] wherever you want … that is why I am afraid to default because the day I default I see I will be punished by going upstairs.” (Client, male, focus group).*

### Implementation challenges

While clients and nurses generally reported positive experiences with POC VL testing (Table S1), some raised concerns about the feasibility of wider implementation in public healthcare facilities. One client thought POC testing may cause delays in clinic flow due to the high number of people within the facility.

*“I don’t think it can work, because it gets full and there will need to be space for people who are waiting [for POC results] and space for people who need to be attended. Where are all these people going to wait? If there are 50 patients waiting and there is another 100 waiting for a doctor or a nurse, there will be a lot of congestion in there.”* (POC male client, 20–30).

In addition, there were concerns about human resources and quality assurance processes required to run decentralized POC machines in public sector clinics. Specifically, one nurse related their experience of the GeneXpert MTB assay, which is based on the same platform as the POC VL assay and has been used for POC tuberculosis (TB) testing at the clinic for several years:

*“The disadvantage is that they only had one official who was doing the job [TB POC testing on GeneXpert]. That was the challenge … sometimes she’s on leave, sometimes she’s off sick, so we would struggle …*. *There were certain issues where the machine would give us kind of questionable results … There were times where the results are not clear and then they would accuse the machine of having had technical errors”* (Healthcare provider).

### Task-shifting to an enrolled nurse as part of differentiated care

Clients who saw an enrolled nurse as part of the STREAM intervention were generally satisfied about the care they received. However, most clients could not tell the difference between an enrolled nurse and a professional nurse.

*“To me the service I received from them was the same, it’s like they were all professionals to me, serious. I can’t even differentiate them.”* (POC male client, 20–30).

Clients who understood the difference between enrolled and professional nurses accepted a differentiated model of care where providing routine treatment was task-shifted to an enrolled nurse, while professional nurses saw clients who required more attention.

*“I can say an enrolled nurse is the one who take bloods and give us medication, the ones that do the easy tasks and then the professional nurse is the one who deals with a person when they are sick, they are almost like doctors.”* (SOC female client, 20–30).

Rather than focussing on a nurse’s level of clinical training, clients emphasized the importance of friendliness, attention and good communication of nurses.

*“When you come to the clinic, the first thing you expect from a nurse is a warm welcome. They should smile, so you can be free to talk to them when you have a problem.”* (Client, female, focus group).

Both enrolled and professional nurses generally felt that task-shifting in facilities with high client numbers would decongest clinics and speed up clinic flows (Table S1).

*“In the government clinics, there are many patients, and many of them are stable. They get delayed because, if for instance, there are two professional nurses in that clinic and the queue is long, we use our staff [enrolled] nurses and the queue will go faster.”* (Professional nurse).

However, enrolled nurses highlighted that professional nurses would be benefited by this. In comparison, professional nurses highlighted that task-shifting to an enrolled nurse would only be acceptable if enrolled nurses are equipped with necessary skills and knowledge to practice safely.

*“The advantages will be for the professional nurses because, uhm, the workload obviously will be less. They won’t be overloaded.” (Enrolled nurse).*

*“They need training. They need to know the antiretrovirals and when we say the patient is stable, they need to understand what we mean by that.”* (Professional nurse).

Professional nurses were worried that enrolled nurses could miss a diagnosis if a patient did not fully report symptoms during a visit.

*“When you do a physical examination you pick up other problems that the participant wasn’t aware of. So an enrolled nurse wouldn’t be able to pick up those things because they are not trained to do that.”* (Professional nurse).

However, enrolled nurses highlighted the benefits of practical experience as part training, which could capacitate them for such responsibilities.

*“Integrate enrolled nurses into hands-on roles with the professional nurses and doctors … to work side by side … If you’ve got us working together on the same thing it makes the work load easier... it educates the enrolled nurse, it gives you more information, you learn a lot when you are working with somebody and then you are seeing how they do things, and what they are doing … It makes it a lot easier.”* (Enrolled nurse).

## Discussion

We found that a differentiated model of care using POC VL testing and task shifting to an enrolled nurse was acceptable to clients and nurses. Practical benefits of POC VL testing included saving time, money and days off work for clients, and increasing the availability of VL results which allowed quicker clinical decisions by nurses. While other studies have investigated perspectives on rapid HIV diagnostic tests [24, 25], there are few qualitative assessments of other POC tests for PLHIV in ART programmes. Healthcare workers in Zimbabwe reported that POC CD4 testing benefited pregnant women with HIV through reduced costs, expedited ART initiation and reduced referrals for CD4 testing to centralized services [16]. In qualitative studies from Uganda and South Africa, healthcare workers felt the benefits of POC testing included reduction in the burden of clinic visits, earlier clinical interventions and the decentralization of testing [26, 27].

We found that POC VL testing helped clients and healthcare workers assess behaviour around adherence and provide instant monitoring of clients’ health. For clients with suppressed VLs, this translated into assessments of doing well, being praised for adherence and seeing an enrolled nurse or being transferred to decentralized care (with easier access to ART). For those with a high VL, it meant being counselled and reflections on how they could change their behaviour to ‘do better’ [28], and the ‘punishment’ of having to continue to collect treatment at the ART clinic, where waiting times are long. The emergence of patient self-regulation through the monitoring of blood test results has been described elsewhere as a ‘disciplinary technology’, where blood results are used to monitor patients’ adherence to treatment and as ‘gatekeepers’ to extended benefits such as CCMDD for those adhering well [28, 29]. Studies of POC CD4 testing in Zimbabwe and South Africa found that clients appreciated receiving quicker results which improved their understanding of CD4 counts [16, 30] and also motivated behavior change [30].

Even though South Africa has a relatively advanced VL testing programme, clients in our study understood CD4 count results better than VL results. This could be due to the longer history of CD4 count testing in South Africa, and also that CD4 results reflect a person’s immune status (i.e. they tell the client something about their ‘self’) [29], whereas VL results are a direct measure of the virus that is more likely to reflect a client’s adherence or the efficacy of their ART. Further work is needed to increase awareness and education around the importance of VL results.

We found that task-shifting from professional nurses to enrolled nurses was acceptable, but ongoing training, supervision and mentoring is likely to be required. Several studies support task-shifting from doctors to appropriately trained professional nurses [20, 31, 32], but there is little research investigating the role of enrolled nurses in primary care ART programmes [33]. Our results highlight that amongst generally well clients, friendliness, respect and good communication were important, rather than the seniority or clinical knowledge of the nurse. In Zambia, a study of preferences for HIV care found that clients were prepared to travel long distances and wait long hours in clinics to be attended to by a friendly healthcare provider [34]. Given the potential for VL monitoring to be used as a ‘disciplinary technology’, it is crucial that results are communicated in a non-judgmental manner that facilitates discussions to encourage adherence [28].

Nurses and clients felt POC VL testing may be challenging in healthcare settings with a high volume of outpatients and insufficient infrastructure. Nurses raised concerns regarding the operation of the POC VL assay in terms of human resources and quality assurance, meaning a simpler, more reliable assay could be an improvement. A systematic review on barriers to implementation of POC testing for HIV found that integration into existing work flows, quality control, diagnostic accuracy, test turnaround times and staff training were major challenges [17]. In South Africa, implementation of rapid HIV diagnosis was impeded by high patient loads and human resource shortages [18], but healthcare workers used multiple strategies to negotiate these challenges [35].

Strengths of our study include the relatively large sample of clients which was broadly reflective of the STREAM study population in terms of demographics and had a broad range of experiences captured by our purposive sampling frame. However, it was a non-randomised sampling approach, and this could introduce bias to the data. Use of interviews and FGDs enabled some data triangulation. Limitations of our study include the single site study, and not all service providers interviewed had experienced POC VL testing. POC testing was performed by a research team, meaning further research is needed to explore implementation in routine healthcare services, where implementation of POC VL testing may prove more challenging. Furthermore, we did not interview other stakeholders who could provide further insights into barriers and facilitators of POC VL testing and task-shifting to enrolled nurses. Lastly, while our sample for this qualitative study was similar demographically to the STREAM study population, we did not obtain quantitative data on the proportion of all STREAM participants who found POC VL testing to be acceptable.

## Conclusions

We found that a differentiated model of care involving POC VL testing and task shifting to enrolled nurses was highly acceptable to clients and nurses. Further implementation studies are needed to identify and understand how to overcome barriers to implementation of POC VL testing, and to develop training programmes to allow enrolled nurses to effectively manage stable clients. If POC VL testing and task shifting to enrolled nurses can be effectively implemented in LMIC ART programmes, they could allow expansion of VL informed differentiated care and contribute towards achieving international treatment targets.

## Supplementary Information

**Additional file 1:.** Topic guides for interviews and focus groups

**Additional file 2: Table S1** Additional quotes highlighting key themes

## Abbreviations

ART: Antiretroviral therapy
CAPRISA: Centre for the AIDS Programme of Research in South Africa
CCMDD: Centralised Chronic Medication Delivery and Distribution Programme
FGD: Focus group discussion
LMICs: Low- and middle-income countries
PLHIV: People living with HIV
POC: Point-of-care
STREAM: Simplified TREAtment and Monitoring study
VL: Viral load
WHO: World Health Organisation
