# Supplementary material for: Acceptability of point-of-care viral load testing to facilitate differentiated care: a qualitative assessment of people living with HIV and nurses in South Africa
Source: BMC Health Serv Res. 2020 Nov 25;20:1081. doi: 10.1186/s12913-020-05940-w (PMC7690121; doi:10.1186/s12913-020-05940-w)
Supplement: Supplementary file 2 — Additional file 2: Table S1 Additional quotes highlighting key themes [file 12913_2020_5940_MOESM2_ESM.docx]

**Table S1. Additional quotes highlighting key themes**

| **Feature** | | **Theme: Practical benefits of POC VL testing** |
| --- | --- | --- |
| Work | | *“As a person who works, I won’t go to work today and take a day off tomorrow when I know my work is not permanent. It would be better to finish with the clinic today and not come back [for results].”* (POC male client, aged 30-40)  *“Sometimes you find that we can’t always ask for a day off.”* (POC female client, aged 30-40)  *“The blood results are very quick as well, you don’t wait for the next appointment, and you just sit and wait for 2 hours. If you are rushing to work, you can come here early and go back to work. There is no need to take the whole day off.* (Client focus group discussion, male*)  *“I believe this method of two hours is second to none, because it takes you as a patient into consideration. [With standard laboratory testing] you will ask to go to the clinic today and ask again the following day as if you work at the clinic. I think there is nothing better than the two hours.”* (POC male client, aged 30-40) |
| Money | | *“Patients that are coming from this facility are coming from faraway places and unfortunately those patients do not have income, so when they get the money to come and be in the facility it was a plus for them to just wait a few hours for the results same day. By the time they go home they know what is happening”* (Standard-of-care healthcare worker^†^)  *“What is good is that you don’t always come to the clinic… and you don’t borrow money today and borrow it tomorrow as well. You only borrow for that day and go to the clinic.”* (POC male client, aged 30-40)  *“The good thing is that you get your blood results on the same day you don’t wait to come back because its costly…. [You] leave knowing that your blood is fine, you don’t have to… this whole thing of going up and down… taking taxis… we don’t all have money to take public transport*” (POC female client, aged 40-50) |
| Time | | *“[POC VL testing] is better, because everything happens here, everything is quick. It saves a lot of time”* (POC male client, aged 30-40)  *“[POC VL testing] helped with [saving] time because it gets full the clinic… here I spent 2 hours.”* (POC female client, aged 20-30) |
| Problems with standard-of-care | | *“I think it would be better taking the viral load and getting the results on the same day rather than coming back [for results] next time… because when I come next time, I have some more issues, so I don’t have time to ask for my bloods… “How is my viral load?” you know? But if you wait, wait for the [POC] results... you get your results.” (Standard-of-care male client, aged 30-40)*  *“I would prefer the method where you get your results same day, because you will know where you have improved and where you need to improve on your health. [With laboratory testing] today you will see this person and when you come back for your next appointment you will see someone who will not explain the results to you… you should be able to see your results the same day. It encourages you because you know...*” (Standard-of-care female client, aged 30-40)  *“It [POC VL testing] can help a lot because waiting for the next month to get your results is not a good thing. That anxiety kills you, you always wonder what the results are going to say, so waiting for a month is not good, introducing this will bring a solution*.” (Client focus group, female) |
| **Theme: Meaning of POC VL results** | | |
| Motivating good adherence behaviour | | *“[POC VL testing results] encouraged me to continue taking my treatment…since they told me it [viral load] was not detected, it means I must continue taking them well”* (POC female client, aged 40-50)  *[POC VL testing] helped me a lot because everything is done on the day, I don’t have to wait for the next month to see my results. Even now I am waiting for my results, I want to see how I have been doing for the past month in terms of taking treatment; whether I am improving or what. I cannot wait for another month, its better if everything is done today.”* (POC male client, aged 20-30) |
| Viral load and CD4 count | | *“I knew there is viral load and CD4 count. I didn’t know what a viral load is, I only knew CD4 count.”* (Client focus group, female)  *“I only knew that my CD4 count is fine and the only important thing to know is a CD4 count until the nurse told me that my viral load is suppressed, and I asked her about it and she explained, then I understood that It should be low and my CD4 count should be high.”* (Client focus group discussion, female)  **Interviewer:** *“Ok, so your CD4 count results they told you that they were 30, what about the viral load results? Did they tell you about them?”*  **Client:** *“I have never paid attention to that, I don’t want to lie I have never paid attention to it” (POC female client, aged 20-30)* |
| **Theme: Differentiated care** | | |
| POC VL testing enabled quick referral | | *“I think it [POC VL testing] helps a lot especially when you are collecting at CCMDD because they will check now and when everything is well they will send you back and when you are not taking your medication they will keep you in the clinic. Also, you won’t have to take bloods today and then come back to see if you will be sent back to CCMDD after two or three months. They will know now.”* (Client focus group discussion, male)  *“They took the bloods and I got my results after 2 hours… and the results explain how you are taking your treatment, whether you need to go to CCMDD …. So, if you don’t take your treatment well they will take you back to the clinic. But if you take your medication well they will transfer you to CCMDD.”* (Client focus group discussion, male) |
| CCMDD as a reward for good adherence | | *“The lady said I am taking my medication well, they will transfer me to CCMDD and then I agreed”* (Client focus group, male)  *“They once told me that if my bloods say otherwise, I will no longer go back to the pharmacy, I will need to be monitored by a doctor at the clinic. Those who remain in the clinic, are the ones that won’t take their medication properly.”* (Client focus group discussion, male) |
| **Theme: Implementation challenges** | | |
| Not feasible at larger scale | | *“I don’t think it’s feasible in a large scale because at the clinic you’d find that you see 60 … ART patients per day, so can you imagine?”* (POC healthcare worker)  **Interviewer***: “If this method [of POC VL testing] was used at a local clinic and you have to wait for the results two hours, how do you think it will be?”*  **Client:** “*There are a lot of people there at the local clinic, you will wait and sometimes wait for more than two hours.”* (POC female client, aged 20-30)  *At the local clinic… they take a lot of people and end up not finishing them in one day... I don’t think they will be able to use the [POC VL testing] system because they still can’t manage the people they currently have. They should add staff as well.* (POC male client, aged 30-40) |
| Maintenance and staffing issues with POC GeneXpert TB testing | | *“The problem was with the machines were overheating, there was no aircon upstairs, so they moved this [GeneXpert MTB] testing to a local hospital, so we don’t do it here anymore,” (*Standard-of-care healthcare worker) |
| **Theme: Task-shifting to an enrolled nurse as part of differentiated care** | | |
| Acceptability | *“I don’t have a problem, I fail to notice the difference between an enrolled nurse and the professional nurse. The enrolled nurse knows her job that is why I didn’t see who the enrolled nurse is because she knows her job.”* (POC female client, aged 40-50)  *“They are all fine, there is nothing different, they were all fine because when I find the enrolled nurse they would be fine and even when I find a professional nurse they would also be fine.”* (POC female client, aged 20-30) | |
| Importance of good communication | *“When you come to the clinic, the first thing you expect from a nurse is a warm welcome. They should smile so you can be free to talk when you have a problem. But if I come in and you’re a busy writing on the file and not even look at me, I won’t talk to you even if I had a problem.”* (Client focus group discussion, female*)*  *“I like too much attention, so I would like them to give me attention. When you talk to me I want to understand you and to make sure that I heard”* (POC female client, aged 20-30) | |
| Training | *“I think it has to start with the training. Yes, uhm, the training should be more, should involve… if they [enrolled nurses] want to deal with patients and treating them, they should go in depth with anatomy and physiology, learn more.* (Professional nurse)  *“For example, if this person is due yearly bloods for their creatinine, viral load and their CD4 then I see them and say move over to the next room to. So, they will need to train, that’s the task shifting we would do maybe to enrolled nurses because that [performing POC testing] is as good as taking bloods [and] enrolled nurses take bloods”* (Professional nurse*)*  *“We have more training than enrolled nurses but when it comes to super-stable participants they know everything that they have to deliver to the patient.”* (Professional nurse) | |
| De-congesting clinics and improving clinic flows | *“It can avoid congestions in the clinics as well, there are a lot of enrolled nurses. If we could do NIMART [ART training] and then pack medication. Because it’s easy, you just take medication and give it to a person, they are super-stable they don’t have problems.”* (Enrolled nurse) | |

* In focus group discussions, it was not possible to determine all demographics for specific participants’ quotes

^†^To protect participant confidentiality, we have not provided complete demographic details of healthcare worker for individual quotes.
